# Development of high-growth influenza H7N9 prepandemic candidate vaccine viruses in suspension MDCK cells

**DOI:** 10.1186/s12929-020-00645-y

**Published:** 2020-04-02

**Authors:** Tsai-Teng Tzeng, Po-Ling Chen, Tsai-Chuan Weng, Shin-Yi Tsai, Chia-Chun Lai, Hsin-I Chou, Pin-Wen Chen, Chia-Chun Lu, Ming-Tsan Liu, Wang-Chou Sung, Min-Shi Lee, Alan Yung-Chih Hu

**Affiliations:** 1 National Institute of Infectious Diseases and Vaccinology, National Health Research Institutes (NHRI), 35 Keyan Road, Zhunan, Miaoli County 35053 Taiwan; 2 Institute of Molecular and Cellular Biology, National Tsing Hua University, Hsinchu, Taiwan; 3 College of Life Science, National Tsing Hua University, Hsinchu, Taiwan; 4 Centers for Disease Control, Ministry of Health and Welfare, Taipei, 689 Taiwan

**Keywords:** H7N9, Highly pathogenic avian influenza, Candidate vaccine virus, Suspension MDCK cells, Chemically defined medium, Reverse genetics

## Abstract

**Background:**

Influenza vaccine manufacturers traditionally use egg-derived candidate vaccine viruses (CVVs) to produce high-yield influenza viruses for seasonal or pandemic vaccines; however, these egg-derived CVVs need an adaptation process for the virus to grow in mammalian cells. The low yields of cell-based manufacturing systems using egg-derived CVVs remain an unsolved issue. This study aimed to develop high-growth cell-derived CVVs for MDCK cell-based vaccine manufacturing platforms.

**Methods:**

Four H7N9 CVVs were generated in characterized Vero and adherent MDCK (aMDCK) cells. Furthermore, reassortant viruses were amplified in adherent MDCK (aMDCK) cells with certification, and their growth characteristics were detected in aMDCK cells and new suspension MDCK (sMDCK) cells. Finally, the plaque-forming ability, biosafety, and immunogenicity of H7N9 reassortant viruses were evaluated.

**Results:**

The HA titers of these CVVs produced in proprietary suspension MDCK (sMDCK) cells and chicken embryos were 2- to 8-fold higher than those in aMDCK cells. All H7N9 CVVs showed attenuated characteristics by trypsin-dependent plaque assay and chicken embryo lethality test. The alum-adjuvanted NHRI-RG5 (derived from the fifth wave H7N9 virus A/Guangdong/SP440/2017) vaccine had the highest immunogenicity and cross-reactivity among the four H7N9 CVVs. Finally, we found that AddaVax adjuvant improved the cross-reactivity of low pathogenic H7N9 virus against highly pathogenic H7N9 viruses.

**Conclusions:**

Our study indicates that cell-derived H7N9 CVVs possessed high growth rate in new sMDCK cells and low pathogenicity in chicken embryo, and that CVVs generated by this platform are also suitable for both cell- and egg-based prepandemic vaccine production.

## Background

The fifth epidemic wave of H7N9 avian influenza in China has raised concerns to public health. Up to May 2019, there have been 1568 human cases, including 616 deaths (mortality rate of approximately 39%) [1]. Although the H7N9 outbreak is under control currently, the newly emerging H7N9 viruses still pose a threat to public health. The H7N9 viruses in the fifth wave have become highly pathogenic avian influenza (HPAI) viruses that bear mutations in the HA gene [2, 3] and display reduced susceptibility to antiviral drugs [4, 5]. Because of the close proximity of Taiwan to the H7N9 outbreak regions, it is crucial for the surveillance and control measure of poultry and migratory birds to be strengthened. Thus, antiviral medicines and H7N9 prepandemic vaccines need to be stockpiled in Taiwan.

Although egg-based manufacturing process is the most common platform to manufacture influenza vaccines in the past 70 years, this platform is labor-intensive and highly dependent on the supply of eggs. Compared with the egg-based production platform, cell-based production platform has more flexibility and scalability in manufacturing process and reduces the potential risk of egg shortage in a pandemic [6–8]. Moreover, cell-based influenza vaccines can avoid the potential risk of egg allergy [7]. Therefore, the cell-based production platform is an alternative manufacturing process to mitigate the shortage of influenza vaccine for pandemic preparedness.

Candidate vaccine viruses (CVVs) released from WHO Essential Regulatory Laboratories (ERLs) are usually derived from eggs and used to produce seasonal and pandemic influenza vaccines. However, the yield of egg-derived CVVs directly cultured in mammalian cells is usually low [9], which increases the cost of production and delays the supply of influenza vaccine during pandemics. To increase the yield of egg-derived CVVs in cell-based production platforms, further adaptation is usually required [10]. We produced inactivated whole-virion H5N1 and H7N9 vaccines in characterized adherent MDCK (aMDCK) cells in a PIC/S GMP bioproduction plant at the National Health Research Institutes (NHRI), Taiwan [11, 12]. However, it took weeks to import the CVVs from ERLs and adapt the CVVs to an aMDCK cell-based platform before vaccine production. For a prompt response to an influenza pandemic, the preparation of cell-derived CVVs is desirable for the rapid production of cell-based pandemic influenza vaccines.

For the preparation of pandemic influenza CVVs, reverse genetic technology is a common method to generate high-growth reassortant viruses using six internal genes derived from A/Puerto Rico/8/34 (PR8) for egg-based manufacturing systems. However, the CVVs generated by using internal genes from PR8 are suitable for egg-based production platform. By using egg-derived CVVs in cell-based platforms, adaptation is usually needed, which takes extra time for preparing production. To shorten the preparation time of vaccine viruses for cell-based manufacturing systems, recent studies used cell-adapted high-growth viruses as master donor viruses (MDVs) [13–15], and synthetic hemagglutinin (HA) and neuraminidase (NA) genes [16, 17] to generate cell-derived CVVs. In addition, for safety issue, the cleavage site of HA protein was modified to -PKGR-, a cleavage site of LPAI H7N9 viruses, and the position 292 of NA protein was also modified to Arginine (R) to increase the sensitivity to NA inhibitor, such as oseltamivir and zanamivir [18]. In this study, we generated cell-derived H7N9 CVVs by reverse genetics (six internal genes derived from an aMDCK cell-adapted H5N1 virus (NIBRG-14) and two attenuated synthetic HA and NA genes). A strategy to enhance the virus yield of CVVs by using the cell-derived CVV technology in our proprietary suspension MDCK (NHRI sMDCK) cells was presented.

## Methods

### Viruses, cells, and medium

The A/Taiwan/1/2017 H7N9 wild-type virus was kindly supplied by the Taiwan CDC. Four H7N9 CVVs were generated using reverse genetics. Vero cells (ATCC CCL-81) were cultivated in VP-SFM medium (GibcoBRL) supplemented with 4 mM glutamine (GibcoBRL), and aMDCK cells (ATCC CCL-34) were cultivated in OptiPro medium (GibcoBRL) supplemented with 4 mM glutamine. After adaptation to serum-free media, Vero and aMDCK cell banks were characterized by Bioreliance (UK), and both cell lines were confirmed to be nontumorigenic. For viral growth, the culture medium was supplemented with 2 μg/mL TPCK-trypsin (Sigma).

### Adaptation of adherent MDCK cells to suspension growth

The proprietary sMDCK cells were cultivated in chemically defined BalanCD® Simple MDCK medium (FUJIFILM Irvine Scientific) supplemented with 4 mM glutamine, and the suspension adaptation process was previously shown in details (PCT Patent No. WO2017072744A1). The adherent MDCK cells were seeded at 5 × 10^5^ cells/mL in spinner flask without microcarriers and culture medium contained 5% (v/v) fetal bovine serum (FBS). Spinner flasks were placed on a stir plate (45–55 rpm) in a 37 °C, humidified incubator with 5% CO_2_. During the period of adaptation, cells in the suspension culture was refreshed with commercial serum-free medium every 3–4 days until the cells grew in suspension (i.e. minimal aggregation) with low proportion of serum. In order to grow the suspension-adapted MDCK (sMDCK) cells without serum, the sMDCK cells were further adapted with several commercial serum-free medium and were frozen with 10% DMSO as a master cell stock. The sMDCK cells were finally adapted into serum-free BalanCD® Simple MDCK medium and were frozen with 10% DMSO as a working cell stock. For routine maintenance in spinner flask, sMDCK cells were inoculated at a concentration of 2 × 10^5^ cells/mL in serum-free BalanCD® Simple MDCK medium, and maximum cell density in suspension cultures are about 1.8 × 10^6^ cells/mL.

### Plasmid construction

Six internal genes derived from aMDCK-adapted NIBRG-14 virus were reverse transcribed using Uni12 primer and amplified with a universal primer set [19]. The HA and NA genes of H7N9 viruses were synthesized (GENEWIZ), and the polybasic cleavage site in HA and position 292 (N2 numbering) of the NA gene were modified (see Table 1). Eight influenza virus genes were cloned into the pHW2000 vector for rescuing CVVs using reverse genetics [20].

**Table 1:** Hemagglutinin cleavage site and NAI resistance marker in wild-type viruses and CVVs

CVVs	Wild-type viruses	Pathogenicity	Modification of hemagglutinin cleavage site		Modification of NAI resistance marker (R292K)	
			Wild type	CVVs	Wild type	CVVs
NHRI-RG3	A/Guangdong/17SF003/2016	HPAI	PEVPKRKRTAR/GLF	PEVPKGR/GLF	K	R
NHRI-RG4	A/Hong Kong/125/2017	LPAI	PEIPKGR/GLF	PEIPKGR/GLF	R	R
NHRI-RG5	A/Guangdong/SP440/2017	HPAI	PEVPKRKRTAR/GLF	PEVPKGR/GLF	K	R
NHRI-RG6	A/Taiwan/1/2017	HPAI	PEVPKRKRTAR/GLF	PEVPKGR/GLF	K	R

Note: *HPAI* highly pathogenic avian influenza, *LPAI* low pathogenic avian influenza, *NAI* neuraminidase inhibitor

### Generation of H7N9 CVVs by reverse genetics

**Fig. 1:**
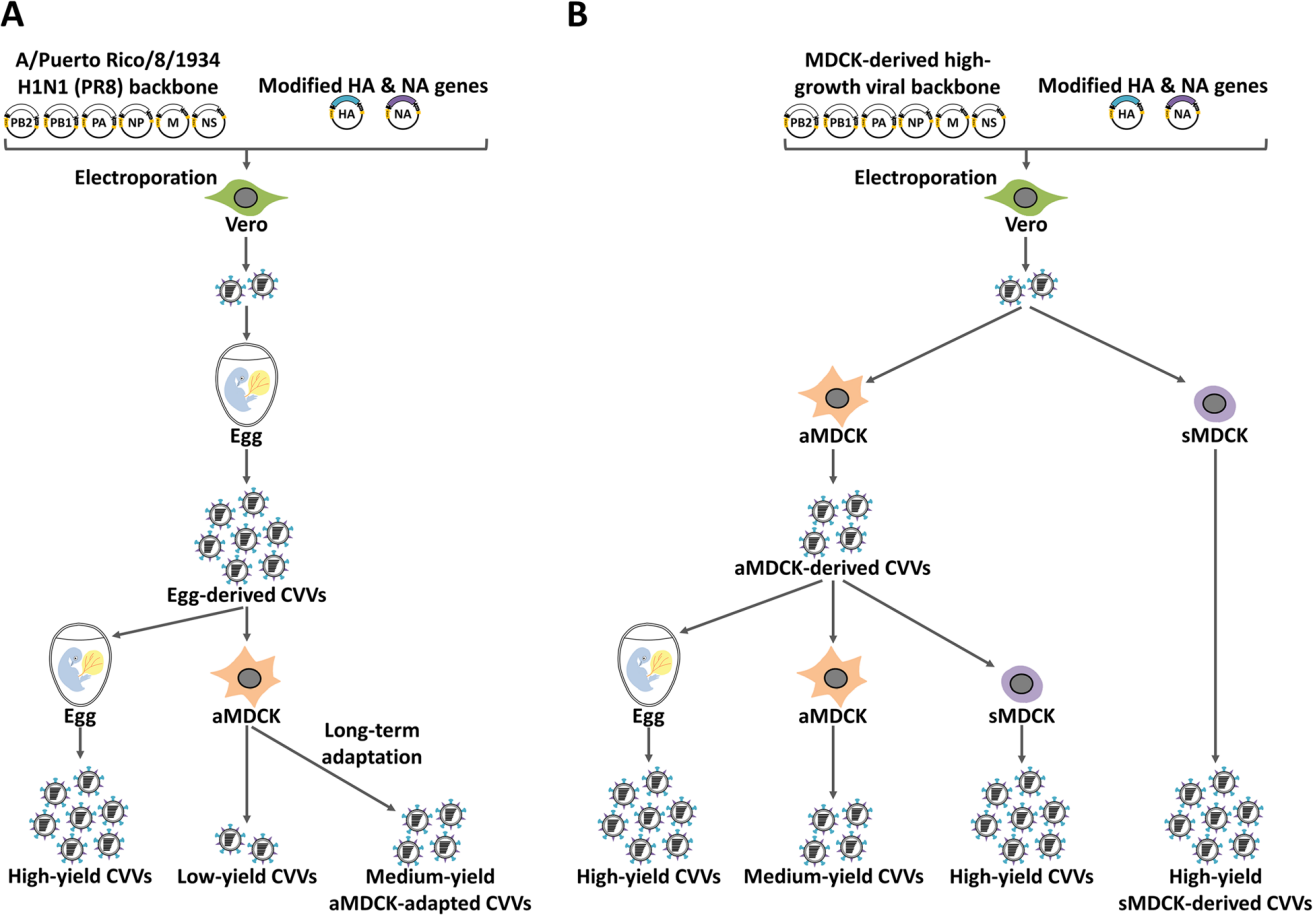
Flowchart for the generation of H7N9 candidate vaccine viruses in serum-free medium by reverse genetics. **a** The development of egg-derived CVVs by reverse genetics using the high-growth PR8 backbone. Egg-derived CVVs are suitable for egg-based manufacturing systems, but their viral yield in cell-based manufacturing systems (aMDCK cells) is usually low. **b** The development of cell-derived CVVs by reverse genetics using a high-growth backbone from aMDCK-adapted NIBRG-14. The cell-derived CVVs grown well in egg- and sMDCK-based manufacturing systems in this study

For safety, all experiments with H7N9 CVVs were conducted in biosafety level 3 (BSL-3) containment approved by the Taiwan CDC. To rescue the 6:2 CVVs, eight plasmids expressing HA, NA, and six internal genes were transfected into Vero cells by electroporation (Fig. 1B). At 4 days posttransfection, virus-containing supernatant (designated V1) was collected and added to aMDCK or sMDCK cells to amplify the rescued viruses (V1aM1 or V1sM1). Viral titers were determined by hemagglutination (HA) and 50% tissue culture infective dose (TCID_50_) assays. The passage history of the reassortant viruses was labeled with the number of passages in the indicated cells (V, Vero cells; aM, adherent MDCK cells; sM, suspension MDCK cells; E, eggs). For example, V1aM3 indicates that the reassortant virus was initially grown in Vero cells, followed by 3 passages in aMDCK cells.

### Evaluation of viral growth properties

A confluent monolayer of aMDCK cells (approximately 2× 10^7^ cells) was grown in a T150 flask and infected with reassortant viruses (V1aM3) at a multiplicity of infection (MOI) of 0.0001. Suspension MDCK cells were grown in a spinner flask. When the cell density of sMDCK cells reached approximately 1.8 × 10^6^ cells/mL from a seeding density of 2.5 × 10^5^ cells/mL, reassortant viruses (V1aM3) were inoculated at an MOI of 0.0001. Finally, the supernatant was collected daily after infection.

### Analysis of genetic stability

To evaluate the genetic stability of CVVs, the reassortant viruses (V1aM3 and V1sM3) were grown in MDCK cells for 4 passages. As mentioned before, the viruses were inoculated to MDCK cells and collected after incubation for 72 h at 35 °C, and the HA and NA genes of the virus stocks (V1aM3 and V1aM7) were sequenced.

### Plaque assay in MDCK cells with or without trypsin

The plaque characteristics of the reassortant viruses (V1aM3) were determined on aMDCK cells. aMDCK cells were grown on 6-well culture plates. Before infection, aMDCK cells were washed with 1× Dulbecco’s phosphate-buffered saline (DPBS) and inoculated with 0.5 mL of diluted viruses in each well. After 60 min of incubation at 35 °C, the cells were washed with 1× DPBS and covered with 3 mL of medium containing 0.3% agarose with or without 1 μg/mL TPCK-treated trypsin. After 72 h of incubation at 35 °C, the cells were fixed with 3.7% formaldehyde and stained with 0.5% crystal violet to visualize the plaques.

### Chicken embryo lethality test

This study protocol was approved by the Institutional Animal Care and Use Committee of NHRI (Protocol No: NHRI-IACUC-107030-A). Ten-day-old chicken eggs (specific antibody-negative eggs) were inoculated with 0.1 mL of 10-fold serially diluted virus (V1aM3) with known TCID_50_ titers. Embryo viability was recorded at 48 h postinfection. The median chicken embryo lethal dose (CELD_50_) was calculated by the method of Reed and Muench [21]. Furthermore, the allantoic fluid of six eggs injected with viruses (V1aM3) diluted 1000-fold was harvested on the third day postinfection (DPI). The HA titer and viral titer of the harvested allantoic fluid were determined by hemagglutination and TCID_50_ assays, respectively.

### Purification for H7N9 reassortant viruses

The H7N9 bulks produced in sMDCK cells were purified according to a previous study [22] with the following modifications. First, 400 mL of the harvested virus was inactivated, and then the cell debris was removed using centrifugation at 1800×g for 30 min. Next, the inactivated virus was further purified using Capto Q and Capto core 700 anion exchange chromatography columns in an AKTA purifier 100 system (GE Healthcare). The flow-through virus solution was diafiltered with PBS using tangential flow filtration with a 100 kDa membrane cassette (Sartorius). Finally, this purified virus bulk was sterilized by using a 0.22 μm filter and stored at 4 °C. The amount of HA antigen in the bulk virus stock was calculated based on the band intensity of the viral protein on a 10% NuPAGE Bis-Tris gel (Thermo Fisher Scientific), and the amount of total viral protein was measured using a Modified Lowry Protein Assay kit (Thermo Fisher Scientific) [23].

### Vaccine preparation and immunization

The mouse study protocol was approved by the Institutional Animal Care and Use Committee of NHRI (Protocol No: NHRI-IACUC-107106-A). Six-week-old female BALB/c mice (*n* = 6 per group) were intramuscularly injected with two doses of vaccine (at day 0 and day 14) containing 0.2 μg of HA antigen and aluminum hydroxide (Brenntag AG) or AddaVax™ (InvivoGen) adjuvant. The amount of alum adjuvant was 30 μg per dose. AddaVax was used according to the manufacturer’s instructions. At day 28, blood samples were collected in serum separator tubes (BD BioSciences). The serum was isolated by centrifugation at 3000 rpm for 10 min and stored at − 20 °C.

### Hemagglutination inhibition (HI) assay

The hemagglutination inhibition (HI) assay was used to assess functional antibodies that inhibit hemagglutination [24]. The standard HA antigen (No. 18/196) and HA antiserum (No. 18/112) for H7N9 (A/Guangdong/17SF003/2016) influenza virus, were purchased from the UK NIBSC.

### Statistical analysis

Statistical data were generated using GraphPad Prism 5 software. HI titers were transformed into logarithmic values and statistical significance between groups was analyzed by one-way analysis of variance (ANOVA) with Newman-Keuls post-test [25].

## Results

### Selection of H7N9 strains for CVV preparation

WHO ERLs usually developed egg-derived CVVs for influenza vaccine production. To evaluate the potential of our cell-derived CVVs, we selected two H7N9 viruses (A/Guangdong/17SF003/2016 and A/Hong Kong/125/2017) that were used by WHO ERLs. Our previous study showed that the acquisition of glycosylation site in HA protein during the adaptation of the NIBRG-268 virus in MDCK cells improved the HA titer and growth efficiency of the influenza H7N9 virus without affecting viral antigenicity [12]. To explore the role of acquired glycosylation in the growth of H7N9 CVV in MDCK cells, we also generated CVVs derived from the A/Guangdong/SP440/2017 and A/Taiwan/1/2017 H7N9 viruses, which acquired potential N-linked glycosylation site in HA protein during evolution. In this study, four H7N9 CVVs were prepared, and their growth rate, genetic stability, biosafety, and immunogenicity were analyzed.

### Growth property of H7N9 reassortant viruses

To increase viral yields in the aMDCK-based production system, we generated cell-derived CVVs by reverse genetics using an aMDCK-adapted NIBRG-14 virus (an H5N1 candidate vaccine virus) an MDV. The HA titers of NHRI-RG5 and NHRI-RG6 in aMDCK cells ranged from 128 to 256 hemagglutination units (HAU)/50 μL (Table 2) and were maintained at a similar level after several passages (V1aM10, unpublished data). In contrast, the HA titers of NHRI-RG3 and NHRI-RG4 showed an increase from 64 HAU/50 μL (V1aM2) to 256 HAU/50 μL (V1aM3) and were maintained at a level similar to that of V1aM10 (unpublished data), which means in the aMDCK-based production system, the aMDCK cell-adapted MDV could not generate high-growth reassortants for all H7N9 viruses and the reassortant viruses may need further adaptations to reach higher titers (Table 2).

**Table 2:** Viral titers of H7N9 CVVs after serial passaging in Vero cells, MDCK cells, and chicken embryonic eggs

CVVs	HA titer (HAU/50 μL) at each passage^a^							
	V1	V1aM1	V1aM2	V1aM3	V1aM3E1	V1sM1	V1sM2	V1sM3
NHRI-RG3	4	64	64	256	2048	512	512	1024
NHRI-RG4	8	64	64	256	2048	512	512	2048
NHRI-RG5	64	256	256	256	2048	2048	2048	2048
NHRI-RG6	64	128	256	256	2048	2048	2048	2048

Note: ^a^Passage history: *V* Vero cells, *aM* adherent MDCK cells, *E* eggs, *sM* suspension MDCK cells. The number indicates the passage number in the indicated cells

Recently, we developed an sMDCK cell line to overcome the problem of scalability in the adherent cell-based manufacturing process for influenza vaccines. To explore the productivity of CVVs in sMDCK cells, the rescued reassortant viruses were also directly inoculated to the cells. The HA titers of sMDCK-derived NHRI-RG3, NHRI-RG4 increased from 64 HAU/50 μL (V1aM1) to 256/512 HAU/50 μL (V1sM1) (Table 2). Similarly, the HA titers of sMDCK-derived NHRI-RG5 and -RG6 showed an 8- to 16-fold increase, compared with their aMDCK-derived counterparts (Table 2). These results suggest that the inoculation of the rescued reassortant virus in sMDCK cells directly improved viral titer by 8- to 16-fold without further adaptation. Interestingly, the MDCK-derived reassortant viruses still have a good growth property in eggs, and could grow to high HA titers in chicken embryos which reaching 2048 HA/50 μL (V1aM3E1, Table 2).

**Fig. 2:**
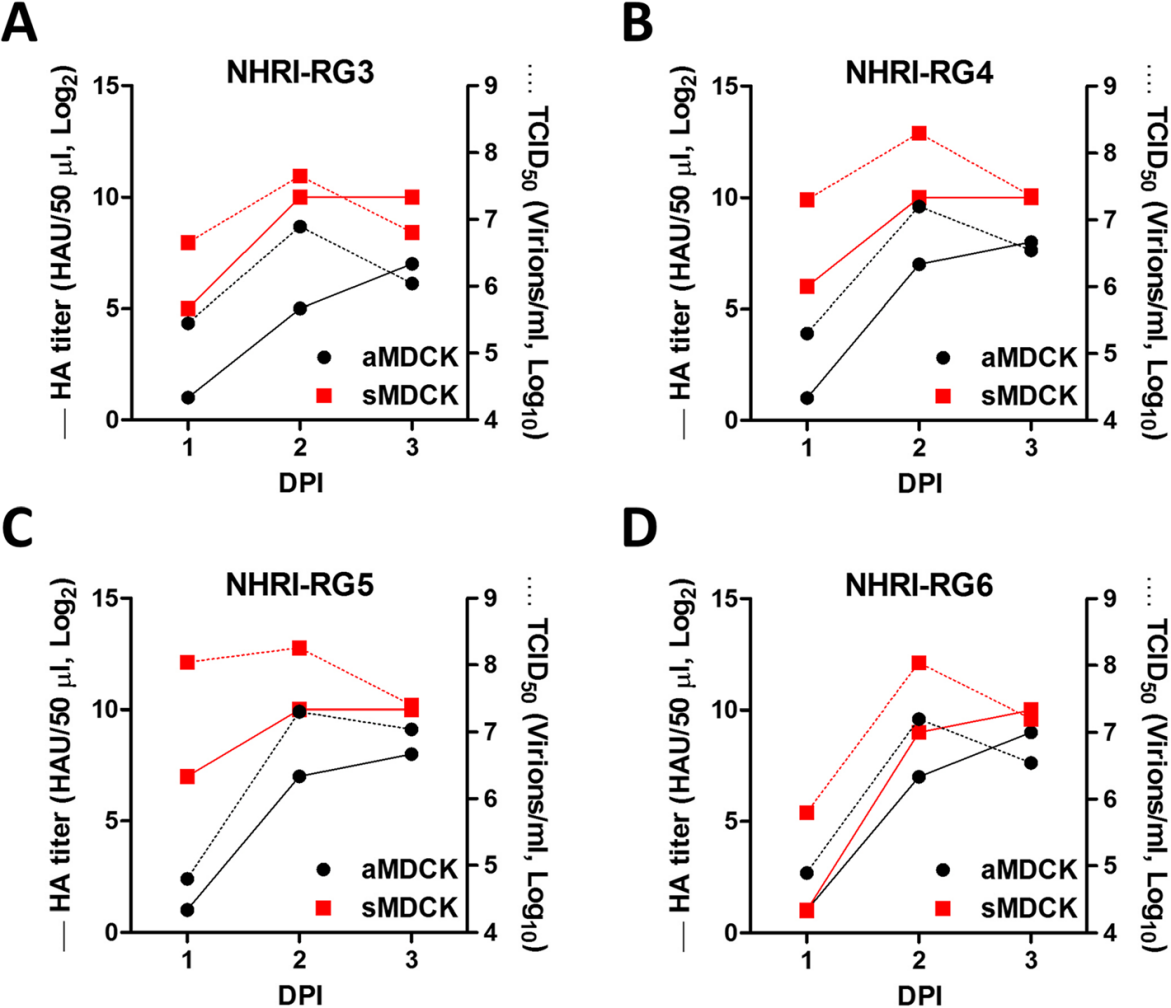
Growth properties of reassortant H7N9 viruses in aMDCK and sMDCK cells. **a** NHRI-RG3 (A/Guangdong/17SF003/2016 H7N9), (**b**) NHRI-RG4 (A/Hong Kong/125/2017 H7N9), (**c**) NHRI-RG5 (A/Guangdong/SP440/2017 H7N9), and (**d**) NHRI-RG6 (A/Taiwan/1/2017 H7N9). Cells were infected with the indicated viruses (V1aM3) at a multiplicity of infection (MOI) of 0.0001 and incubated at 35 °C. Cell culture supernatants were collected at 1, 2, and 3 days postinfection (DPI). The HA titer (solid line) and viral titer (dashed line) of the collected supernatants were determined by hemagglutination and TCID_50_ assays, respectively

To evaluate the antigen yield of CVVs in aMDCK and sMDCK cells, we analyzed the viral growth kinetic of H7N9 CVVs. The H7N9 CVVs from sMDCK cells showed an 4-fold increase in HA titers when grown in sMDCK cells (the solid line in Fig. 2) compared with that from aMDCK cells. Similarly, the infectious titers of CVVs at day 1, 2, and 3 after infection were shown in Fig. 2 (dashed line), and the peak viral titers of each CVVs were observed by day 2. The infectious titers of CVVs in sMDCK cells were higher than that in aMDCK cells at day 2 after infection. Taken together, these data indicate that the sMDCK-based production platform has commercial potential for influenza vaccines.

### Genetic stability of the HA and NA genes in H7N9 CVVs

To evaluate the genetic stability of the HA and NA genes, aMDCK and sMDCK cells were inoculated with H7N9 CVVs for multiple passages and the sequence of the HA and NA genes in passage 3 and 7 was analyzed. The NA sequences were not changed up to passage 7 and the introduced K292R in NA (Table 1) was retained. Importantly, the sequence of the modified HA cleavage site remained unchanged as a monobasic amino acid in passage 3 and 7, suggesting that H7N9 CVVs remained low pathogenic avian influenza (LPAI) up to passage 7.

As mentioned previously, there was an increase in the HA titers of NHRI-RG3 and NHRI-RG4 at the V1aM3 and V1sM3 passages (Table 2). The sequencing results showed that an HA-I120T mutation (H7 numbering) appeared in NHRI-RG3 during the V1aM3 passage, and this mutation became dominant at the V1aM7 passage (Table 3). There was also a mutation, HA-A151T, in NHRI-RG4 at the V1aM3 passage, and this mutation was maintained at the V1aM7 passage (Table 3). No mutation was identified in internal genes (PB1, PB2, PA, NP, NS, and M genes) of H7N9 CVVs propagated in aMDCK or sMDCK cells, hence, these HA mutations in NHRI-RG3 and NHRI-RG4 may be the cause of increased HA titer. In terms of NHRI-RG3 and NHRI-RG4 at the V1sM3 and V1sM7 passage, several amino acid substitutions in HA were found in NHRI-RG3 (I120T, K163R, and A292T) and NHRI-RG4 (I120T, I421V, and I421M) (Table 3). Interestingly, I120T substitution was found in NHRI-RG3 (V1aM3 and V1sM3) and NHRI-RG4 (V1sM4). Although the role of other substitutions remains unclear, I120T substitution could be the main factor for the improved viral growth of these viruses.

**Table 3:** Amino acid substitutions in the HA protein of H7N9 CVVs propagated in aMDCK or sMDCK cells

CVVs	V1aM3^a^	V1aM7^a^	V1sM3^a^	V1sM7^a^
NHRI-RG3	I120T/I^b^	I120T	I120I/T^b^, G209E/G^b^	I120T, K163R, A292T/A^b^
NHRI-RG4	A151T	A151T	I120I/T^b^	I120T/I^b^, I421I/V/M^b^
NHRI-RG5	No change	No change	No change	No change
NHRI-RG6	No change	No change	No change	No change

Note: ^a^Passage history: *V* Vero cells, *aM* adherent MDCK cells, *sM* suspension MDCK cells. The number indicates the passage number in the indicated cells

^b^Mixed amino acid residues were detected

### Trypsin-dependent plaque formation

**Fig. 3:**
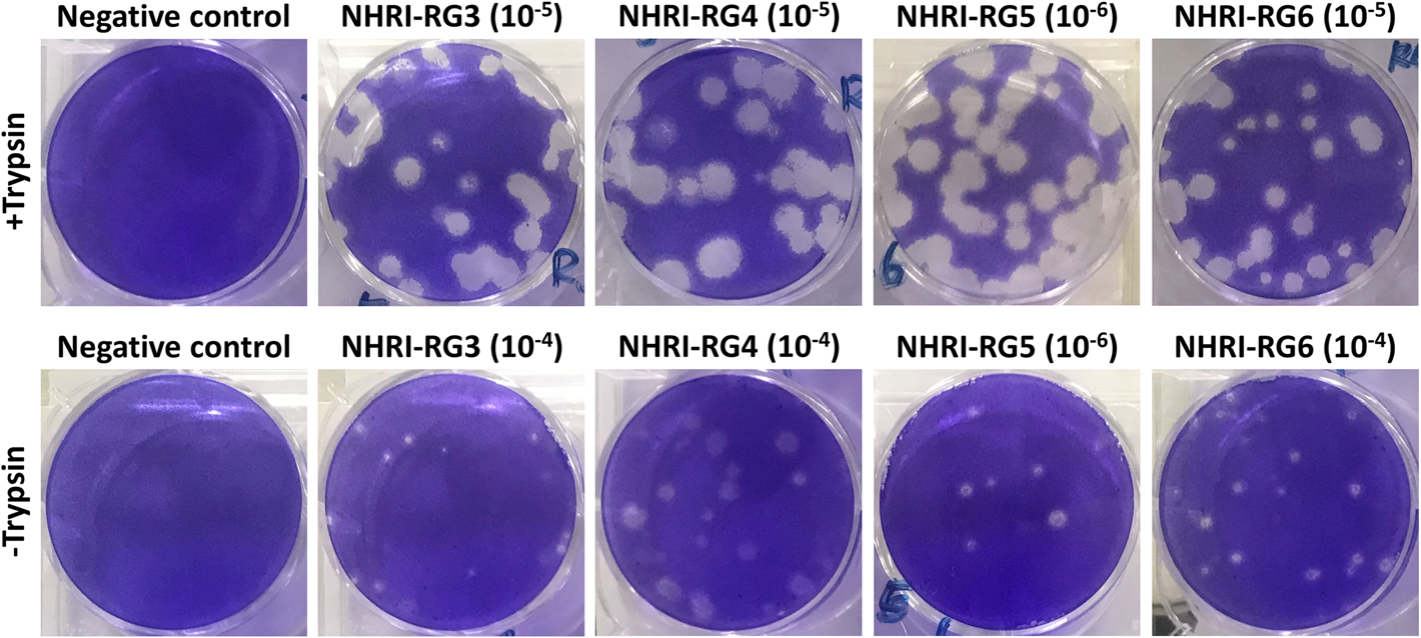
Plaque-forming ability of H7N9 reassortant viruses in MDCK cells with or without trypsin. The indicated viruses were diluted to the value shown in parentheses and MDCK cells were inoculated with the diluted viruses. The negative control indicates MDCK cells treated with DPBS

Trypsin-dependent plaque formation is a feature of LPAI and attenuated CVVs. A previous study showed that HPAI H7N9 has a trypsin-independent plaque-forming ability [2], which is conferred by the polybasic cleavage site in HA. The modified HA cleavage site of H7N9 CVVs was similar to that of LPAI, so we examined whether the plaque-forming ability of H7N9 CVVs is trypsin-dependent. In the presence of trypsin, H7N9 CVVs exhibited viral titers ranging from 10^8.20^ to 10^9.05^ PFU/mL (Table 4). In the absence of trypsin, the plaque-forming ability of H7N9 CVVs was reduced 10-fold (Table 4), and the plaque size was reduced (Fig. 3). These results indicate that the plaque-forming ability of all H7N9 CVVs is trypsin-dependent, which is consistent with the sequence of the monobasic cleavage site in HA.

**Table 4:** Plaque formation of H7N9 CVVs

CVVs	With trypsin	Without trypsin
NHRI-RG3	10^8.34^ PFU/mL	10^7.07^ PFU/mL
NHRI-RG4	10^8.20^ PFU/mL	10^7.45^ PFU/mL
NHRI-RG5	10^9.05^ PFU/mL	10^8.10^ PFU/mL
NHRI-RG6	10^8.50^ PFU/mL	10^7.22^ PFU/mL

Note: *PFU* plaque-forming units

### Biosafety evaluation by using chicken embryonic eggs

Before vaccine manufacture, the biosafety of CVVs is usually evaluated in eggs, chickens, and ferrets [26]. Previous study showed that chicken embryo lethality of CVVs and HPAI viruses is consistent with their pathogenicity in chickens and ferrets [27]. Because our current facility is not equipped for experiments in chickens and ferrets, we tested the pathogenicity of H7N9 CVVs in chicken embryonic eggs. HPAI H7N9 virus (A/Taiwan/1/2017) was used as a positive control, and its CELD_50_ was 10^–1.17^ TCID_50_ (Table 5). In contrast, the CELD_50_ of H7N9 CVVs in this study was more than 10^6.65^ TCID_50_ (Table 5), suggesting that the CVVs were attenuated with low levels of pathogenicity.

**Table 5:** Biosafety assessment of H7N9 CVVs

CVVs	CELD_**50**_^a^
Wild-type H7N9 (A/Taiwan/1/2017)	10^–1.17^ TCID_50_
NHRI-RG3	> 10^6.65^ TCID_50_
NHRI-RG4	> 10^7.04^ TCID_50_
NHRI-RG5	> 10^7.65^ TCID_50_
NHRI-RG6	> 10^6.65^ TCID_50_

Note: ^a^CELD_50_: median chicken embryo lethal dose; expressed as median tissue culture infectious dose (TCID_50_)

### Antigenicity and immunogenicity of the 5th wave H7N9 vaccines

The major differences in the HA1 sequences among the selected H7N9 CVVs included I38T, T112P, I120T, K130R, K164E, L217Q, and I317V (Additional file 1: Table S1). To investigate the antigenicity and immunogenicity of the CVVs, H7N9 CVVs were produced in sMDCK cells and purified by using flow-through chromatography. Electron microscopy images revealed that the four purified H7N9 CVVs had a spherical shape with clear spike structures on the surface (Additional file 2: Fig. S1). The antigenicity of H7N9 reassortant viruses was determined by HI assay using A/Guangdong/17SF003/2016-like standard serum. The HI assay results revealed that the antigenicity of NHRI-RG3, RG4, and RG5 (V1aM3sM1) was similar to that of A/Guangdong/17SF003/2016-like standard antigen (Table 6). The sequencing results showed that NHRI-RG3, RG4, and RG5 at V1aM3sM1 passage had HA-I120T or HA-A151T substitutes without other mutation after an sMDCK passage (unpublished data). Hence, these results indicated that HA-I120T and HA-A151T substitutes did not change viral antigenicity. Notably, the HI titer of NHRI-RG6 was 4-fold lower than that of NHRI-RG3, RG4, and RG5, as well as standard antigen, suggesting that the antigenicity of NHRI-RG6 was different with those viruses (Table 6). NHRI-RG6 (derived from A/Taiwan/1/2017) had several differences in HA1 amino acid sequence compared with A/Guangdong/17SF003/2016 H7N9 virus (Additional file 1: Table S1), which could be the reason for the antigenic difference between NHRI-RG3 and -RG6.

**Table 6:** HI activity of sheep standard serum against inactivated H7N9 bulks

CVVs	HI titer
Standard antigen (No. 18/196)	640
NHRI-RG3	640
NHRI-RG4	640
NHRI-RG5	640
NHRI-RG6	160

Note: Inactivated H7N9 bulks were prepared in sMDCK cells (V1aM3sM1)

**Fig. 4:**
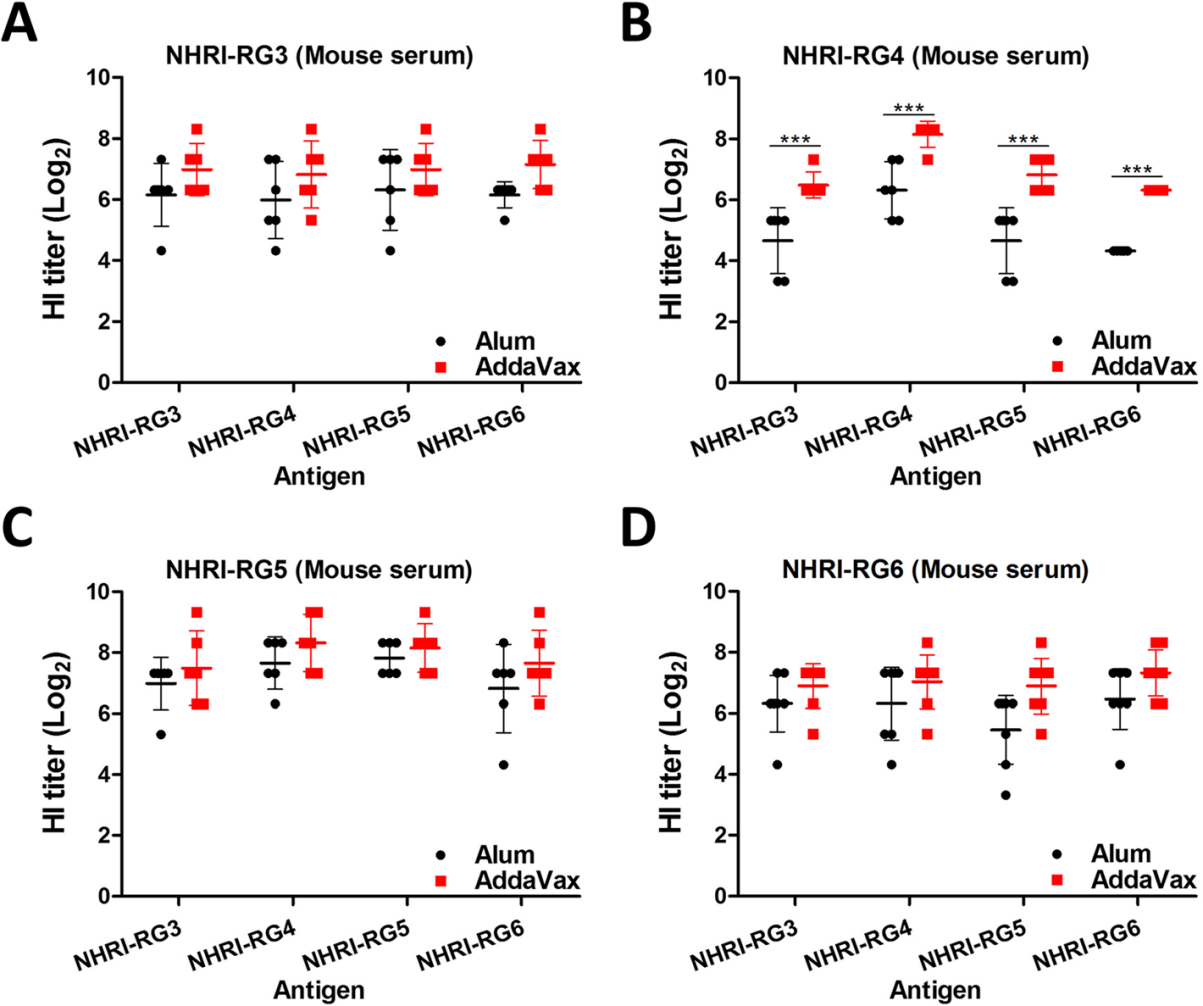
HI activity of mouse serum against inactivated H7N9 CVVs. Each H7N9 bulk (NHRI-RG3, NHRI-RG4, NHRI-RG5, and NHRI-RG6) was mixed with alum or AddaVax adjuvant, and administered to BALB/c mice intramuscularly on day 0 and day 14. The reactivity of mouse serum against inactivated NHRI-RG3, NHRI-RG4, NHRI-RG5, and NHRI-RG6 CVVs (Antigen) was examined by an HI assay, using the serum collected on day 28. The line indicates the geometric mean with a 95% confidence interval. The log_2_-transformed HI titers were analyzed by ANOVA with Newman-Keuls post-test (*n* = 6 mice for NHRI-RG3, NHRI-RG4 and NHRI-RG5 groups; *n* = 7 mice for NHRI-RG6 group). Significant differences between alum- and AddaVax-adjuvanted vaccines are indicated by ***, *p* < 0.001

Because squalene-based adjuvant (AddaVax) can improve immune response and antibody cross-reactivity [28], we evaluated the immune response of alum- and AddaVax-adjuvanted H7N9 vaccines. The antisera of immunized mice were analyzed using an HI assay against homologous and heterologous H7N9 viruses. In the alum-adjuvanted groups, the geometric mean HI titers of NHRI-RG3, NHRI-RG4, NHRI-RG5, and NHRI-RG6 vaccines against the homologous virus were 71.3, 80.0, 226.3, and 88.3, respectively (Fig. 4), and the NHRI-RG3, −RG5 and -RG6 vaccines induced a similar level of antibodies against homologous and heterologous H7N9 viruses (Fig. 4A, C, and D). However, the NHRI-RG4 vaccine elicited statistically lower cross-reactive antibody (2- to 4-fold lower) against heterologous H7N9 viruses (NHRI-RG3, RG5 and RG6) than homologous H7N9 virus (NHRI-RG4) (Fig. 4B). In the AddaVax-adjuvanted groups, the geometric mean HI titers of NHRI-RG3, NHRI-RG4, NHRI-RG5, and NHRI-RG6 vaccines against the homologous virus were 127.0, 285.1, 285.1, and 160.0, respectively (Fig. 4). Compared with alum adjuvant, the AddaVax adjuvant slightly enhanced the immunogenicity of the NHRI-RG3, NHRI-RG5, and NHRI-RG6 vaccines (Fig. 4A, C, and D) and significantly improved the immunogenicity of the NHRI-RG4 vaccine against both homologous and heterologous viruses (Fig. 4B). These results indicate that NHRI-RG5 has the highest immunogenicity among all H7N9 CVVs, and the low immunogenicity and cross-reactivity of NHRI-RG4 vaccine may be improved using the AddaVax adjuvant.

## Discussion

The manufacturing of vaccines for emerging pandemic influenza usually takes months with multiple processes, including the generation of high-growth CVVs, viral growth optimization, the evaluation of antigenicity and pathogenicity, and so on [26, 29, 30]. However, our experiences show that the use of egg-derived CVVs in cell-based manufacturing systems could prolong the time to final vaccine production because of the paperwork associated with CVV importation and the viral adaptation in cells (Fig. 1A). These processes may take an additional 2–4 months. In this study, we developed cell-derived CVVs using reverse genetics and generated high-yield CVVs in the sMDCK-manufacturing system (Fig. 1B). This approach could help save time by omitting the time of CVV importation and the optimization of viral growth in cells. Although a 3-passage adaptation to aMDCK cells was required to optimize the growth of NHRI-RG3 and NHRI-RG4 CVVs (Table 2), our results showed that the direct inoculation of the rescued reassortant virus in sMDCK cells improves virus growth without requiring further adaptation (right part in Fig. 1B).

Several suspension MDCK cell lines have also been developed to facilitate cell-based manufacturing processes [31–33]. Similar to these studies, the productivity of influenza viruses in sMDCK cells was higher than that in aMDCK cells (Fig. 2). The differences between sMDCK and aMDCK culture systems include cultivation conditions and the characteristics of the cell line. We found that the cell-specific productivity of viral particles (virions/cell) in the sMDCK culture system was 1.9- to 3.5-fold higher than that in the aMDCK culture system (Supplementary Table S2). A previous study also showed that the HA production capability (HAU/10^6^ cells) of suspension MDCK cells is remarkably higher than that of adherent MDCK cells [31]. Therefore, we assume that the high yield of influenza virus in the sMDCK culture system does not depend entirely on cell density. To further explore the potential mechanism by which influenza virus growth is increased in sMDCK cells, we will examine the transcriptome differences between sMDCK and aMDCK cells.

Previous studies showed that N-linked glycosylation at N123 and N149 sites of the H7 protein enhances the viral growth, which may be related to the influence on receptor binding [34, 35]. Similarly, we found that CVVs (NHRI-RG5 and NHRI-RG6) with these N-linked glycosylation sites had 4-fold higher HA titers than NHRI-RG3 and NHRI-RG4 at passages V1aM1 and V1aM2 (Table 2). In particular, the HA titers of NHRI-RG3 and NHRI-RG4 at passage V1aM3 reached levels similar to those of NHRI-RG5 and NHRI-RG6, which also have amino acid substitutions in the HA protein, including an I120T mutation in NHRI-RG3 and an A151T mutation in NHRI-RG4. The HA-I120T and HA-A151T mutations could potentially induce N-linked glycosylation of the HA protein at N118 (which is the same as NHRI-RG6) and N149 (which is the same as NHRI-RG5), respectively. The N-linked glycosylation at N118 (NHRI-RG3 and NHRI-RG6 and N149 (NHRI-RG4 and NHRI-RG5) in the HA protein has been shown by HPLC-MS/MS (Additional file 3: Fig. S2 and Additional file 4: Fig. S3). Based on previous studies [34, 35] and our findings, the acquired N-glycosylation site at N118 and N149 may be the reason for the improved HA titer of NHRI-RG3 and NHRI-RG4 (V1aM3), respectively, and these N-linked glycosylations at N118 and N149 did not cause any antigenic change in NHRI-RG3 and NHRI-RG4 (Table 6). Notably, the S118N mutation in the HA protein occurred gradually during evolution and became dominant in the 4th and 5th wave H7N9 viruses isolated from human, avian and environmental samples (Additional file 5: Fig. S4A), but the I120T mutation in the HA protein was found only in human samples (Additional file 5: Fig. S4B). Therefore, it is possible that N-linked glycosylation at N118 and N149 increases the growth efficiency of the H7N9 virus in mammalian cells. In the future, it could be an alternative to generate high-growth CVVs for emerging influenza H7N9 virus via introducing HA-I120T or HA-A151T substitutions, because it is acceptable to introduce special amino acid into the HA protein of influenza CVVs without affecting its antigenicity and pathogenicity [36].

Based on genetic and antigenic stability of influenza viruses, a suspension MDCK cell line, MDCK33016PF, has been considered as a suitable platform for the isolation and preparation of influenza vaccine seed viruses, especially H3N2 and B/Yamagata viruses [37]. The sequencing results of eight segments from NHRI-RG5 and -RG6 was quite stable in sMDCK cells after at least serial 7 passages, but several adaptive mutations, I120T, K163R, A292T, I421V, and I421M, were found in NHRI-RG3 and -RG4 after serial 3 passages and required for improving growth efficiency. Although the role of each amino acid substitution in HA remains unclear, these results suggested that the genetic stability of H7N9 CVVs in sMDCK cells is strain-dependent. Further study is still required to explore the genetic and antigenic stability of various influenza viruses after serial passages in sMDCK cells and the potential of sMDCK cells to isolate and prepare influenza vaccine seed viruses.

Based on the WHO guideline to develop and produce pandemic influenza vaccines [26], this study conducted strict safety testing procedures to prove the pathogenicity of developed CVVs, including genetic stability, trypsin-independent plaque-forming ability, and biosafety evaluation. This study revealed that sMDCK-derived high-growth H7N9 CVVs were attenuated with low levels of pathogenicity. Previous study using chicken embryos lethality test showed that reassortant H5N1 CVVs lacks pathogenicity compared with wild-type HPAI viruses, which are consistent with the safety test in ferrets and chickens [27]. According the WHO guideline [26, 38], the safety study should be conducted in ferrets before the CVVs are released to vaccine manufacturers with BSL-2 enhanced containment. This study aims to develop high-growth influenza CVVs in suspension MDCK cells before vaccine manufacturing; hence, we will conduct ferret study in the future before the CVVs are used as vaccine seed viruses.

Although a WHO report showed that the antigenicity of LPAI H7N9 viruses is distinct from that of emerging HPAI H7N9 viruses [39], a previous study illustrated that the AddaVax- adjuvanted LPAI H7N9 vaccine conferred efficient protection against HPAI H7N9 virus infection in a ferret challenge model [40]. Consistently, we found that alum-adjuvanted NHRI-RG4 (LPAI) vaccine induced antibodies with low cross-reactivity against HPAI H7N9 viruses (NHRI-RG3, NHRI-RG5, and NHRI-RG6), but this low cross-reactivity was improved by the use of the AddaVax adjuvant, an MF-59-like adjuvant (Fig. 4B). This improvement has been reported in MF-59-adjuvanted H1N1 vaccines [41]. Interestingly, the antigenicity of NHRI-RG6 was distinct from A/Guangdong/17SF003/2016-like antigen (Table 6), but alum- and AddaVax- adjuvanted HPAI H7N9 vaccines (NHRI-RG3, NHRI-RG5, and NHRI-RG6) could elicit high cross-reactive antibody response to each other (Fig. 4). We also found that AddaVax adjuvant inclusion enhanced the immunogenicity of NHRI-RG4 (Fig. 4B), consistent with the dose-sparing effect of AddaVax adjuvant on the H7N9 vaccine in mice [28]. These findings demonstrate that squalene-based adjuvants have the potential to improve H7N9 vaccine efficacy by increasing cross-reactivity and immunogenicity.

## Conclusions

In summary, we used 6 internal genes of an aMDCK cell-adapted MDV and synthetic HA and NA genes to generate influenza H7N9 reassortant vaccine viruses by establishing reverse genetics. We further found that these cell-derived CVVs have high growth rates in sMDCK cells and demonstrated that four cell-derived H7N9 CVVs have a trypsin-dependent plaque-forming ability and no lethality in chicken embryos. To shorten the preparation time of CVVs for production, it might be ideal to directly establish sMDCK cell-derived CVVs, which could improve the process of CVV preparation by eliminating the use of aMDCK cells. Moreover, the comparison of immunogenic and antigenic properties among the four H7N9 CVVs showed that NHRI-RG5 is the most suitable for the production of prepandemic vaccines. In conclusion, combining the two plaforms (sMDCK and reverse genetics) could significantly improve efficiency and productivity of manufacturing influenza H7N9 vaccines for pandemic preparedness.

## Supplementary information

**Additional file 1: Table S1.** Major differences in the HA1 amino acid sequence of selected reassortant H7N9 viruses.

**Additional file 2: Table S2.** Cell-specific productivity of MDCK cells.

**Additional file 3: Fig. S1.** EM images of H7N9 bulks. sMDCK-derived H7N9 reassortant viruses were purified, viral particles were negatively stained with 2% UA, and the images were captured using EM.

**Additional file 4: Fig. S2.** Identification of N-linked glycosylation at the N118 residue on hemagglutinin by liquid chromatography-tandem mass spectrometry. N-linked glycosylation was identified by liquid chromatography-tandem mass spectrometry, as described in Additional file 6. Tandem mass spectra (MS2) of ESGGIDKEPMGFTYNGTR (m/z 653.96, + 3) derived from the trypsin-digested purified H7N9 bulks, (A) NHRI-RG3 and (B) NHRI-RG6. N# represents the deamidated asparagine which indicates that the N118 residue is glycosylated in the original hemagglutinin protein.

**Additional file 5: Fig. S3.** Identification of N-linked glycosylation at the N149 residue on hemagglutinin by liquid chromatography-tandem mass spectrometry. N-linked glycosylation was identified by liquid chromatography-tandem mass spectrometry, as described in Additional file 6. Tandem mass spectra (MS2) of WLLSNTDNATFPQMTK (m/z 934.44, + 2) derived from the trypsin-digested purified H7N9 bulks, (A) NHRI-RG4 and (B) NHRI-RG5. N# represents the deamidated asparagine which indicates that the N149 residue is glycosylated in the original hemagglutinin protein.

**Additional file 6: Fig. S4.** Evolution of the hemagglutinin N118 glycosylation site in H7N9 viruses from the 1st to 5th epidemic wave. Temporal pattern of S118N (A) and I120T (B) mutations in H7N9 hemagglutinin from human, avian and environmental samples. HA protein sequences were collected and analyzed as described in Additional file 6.

**Additional file 7:** Supplemental materials and methods

## Data Availability

The data that support the findings of this study are available from the corresponding author upon request.

## Abbreviations

aMDCK: Adherent MDCK cells
CVVs: Candidate vaccine viruses
CELD_50_: Median chicken embryo lethal dose
E: Eggs
HPAI: Highly pathogenic avian influenza
HA: Hemagglutinin
HAU: Hemagglutination units
LPAI: Low pathogenic avian influenza
MDVs: Master donor viruses
NA: Neuraminidase
V: Vero cells
DPI: Day post infection
TCID_50_: Median tissue culture infectious dose
sMDCK: Suspension MDCK cells
