# Supplementary material for: Development of high-growth influenza H7N9 prepandemic candidate vaccine viruses in suspension MDCK cells
Source: J Biomed Sci. 2020 Apr 2;27:47. doi: 10.1186/s12929-020-00645-y (PMC7115086; doi:10.1186/s12929-020-00645-y)
Supplement: Supplementary file 1 — Additional file 1: Table S1. Major differences in the HA1 amino acid sequence of selected reassortant H7N9 viruses. [file 12929_2020_645_MOESM1_ESM.pdf]

Additional file 1

Table S1. Major differences in the HA1 amino acid sequence of selected reassortant H7N9 viruses

		Type of amino acid at the specific HA1 position*										
H7N9 viruses	GISAID accession No.	38	112	118	120	130	163	164	217	261	292	317
A/Anhui/1/2013 (LPAI)	EPI_ISL_138739	I	A	S	I	R	R	K	L	G	A	I
A/Guangdong/17SF003/2016 (HPAI)	EPI_ISL_249309	T	P	N	I	R	K	E	Q	R	A	V
A/Hong Kong/125/2017 (LPAI)	EPI_ISL_259269	I	T	N	I	K	R	K	L	G	A	I
A/Guangdong/SP440/2017 (HPAI)	EPI_ISL_256109	T	P	N	I	R	K	E	Q	R	A	V
A/Taiwan/1/2017 (HPAI)	EPI_ISL_248778	T	P	N	T	R	R	E	Q	G	T	V

HPAI, Highly pathogenic avian influenza; LPAI, low pathogenic avian influenza.

*H7 numbering.

HA protein sequences were collected and analyzed as described in Additional file 6.
