# Supplementary material for: Development of high-growth influenza H7N9 prepandemic candidate vaccine viruses in suspension MDCK cells
Source: J Biomed Sci. 2020 Apr 2;27:47. doi: 10.1186/s12929-020-00645-y (PMC7115086; doi:10.1186/s12929-020-00645-y)
Supplement: Supplementary file 2 — Additional file 2: Table S2. Cell-specific productivity of MDCK cells. [file 12929_2020_645_MOESM2_ESM.docx]

**Additional file 2**

**Table S2. Cell-specific productivity of MDCK cells.**

|  | aMDCK | | |  | sMDCK | | |
| --- | --- | --- | --- | --- | --- | --- | --- |
|  | TCID_50_/mL* | Cells/mL | Virions/cell |  | TCID_50_/mL* | Cells/mL | Virions/cell |
| NHRI-RG3 | 7.80E+06 | 5.0E+05/mL | 15.6 |  | 4.45E+07 | 1.8E+06/mL | 24.7 |
| NHRI-RG4 | 1.60E+07 | 5.0E+05/mL | 32 |  | 1.99E+08 | 1.8E+06/mL | 110.6 |
| NHRI-RG5 | 1.99E+07 | 5.0E+05/mL | 39.8 |  | 1.77E+08 | 1.8E+06/mL | 98.3 |
| NHRI-RG6 | 1.60E+07 | 5.0E+05/mL | 32 |  | 1.12E+08 | 1.8E+06/mL | 62.2 |

*The value of virus titer (TCID_50_/mL) of CVVs at day 2 after infection was shown.
