# Supplementary material for: Development of high-growth influenza H7N9 prepandemic candidate vaccine viruses in suspension MDCK cells
Source: J Biomed Sci. 2020 Apr 2;27:47. doi: 10.1186/s12929-020-00645-y (PMC7115086; doi:10.1186/s12929-020-00645-y)
Supplement: Supplementary file 3 — Additional file 3: Fig. S1. EM images of H7N9 bulks. sMDCK-derived H7N9 reassortant viruses were purified, viral particles were negatively stained with 2% UA, and the images were captured using EM. [file 12929_2020_645_MOESM3_ESM.pdf]

Additional file 3

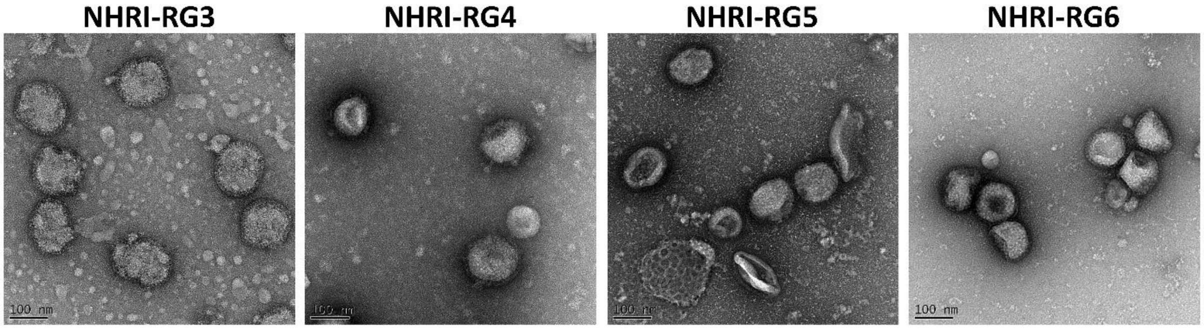

Figure S1. EM images of H7N9 bulks.

sMDCK-derived H7N9 reassortant viruses were purified, viral particles were negatively stained with 2% UA, and the images were captured using EM.
