# Supplementary material for: Development of high-growth influenza H7N9 prepandemic candidate vaccine viruses in suspension MDCK cells
Source: J Biomed Sci. 2020 Apr 2;27:47. doi: 10.1186/s12929-020-00645-y (PMC7115086; doi:10.1186/s12929-020-00645-y)
Supplement: Supplementary file 4 — Additional file 4: Fig. S2. Identification of N-linked glycosylation at the N118 residue on hemagglutinin by liquid chromatography-tandem mass spectrometry. N-linked glycosylation was identified by liquid chromatography-tandem mass spectrometry, as described in Additional file 6. Tandem mass spectra (MS2) of ESGGIDKEPMGFTYNGTR (m/z 653.96, + 3) derived from the trypsin-digested purified H7N9 bulks, (A) NHRI-RG3 and (B) NHRI-RG6. N# represents the deamidated asparagine which indicates that the N118 residue is glycosylated in the original hemagglutinin protein. [file 12929_2020_645_MOESM4_ESM.pdf]

Additional file 4

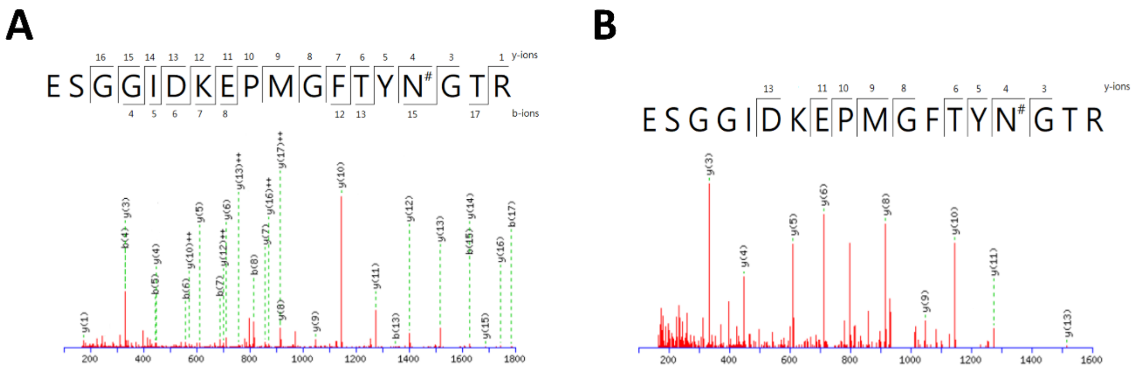

Figure S2. Identification of N-linked glycosylation at the N118 residue on hemagglutinin by liquid chromatography-tandem mass spectrometry.

N-linked glycosylation was identified by liquid chromatography-tandem mass spectrometry, as described in Additional file 6. Tandem mass spectra (MS2) of ESGGIDKEPMGFTYNGTR (m/z 653.96, +3) derived from the trypsin-digested purified H7N9 bulks, (A) NHRI-RG3 and (B) NHRI-RG6. N# represents the deamidated asparagine which indicates that the N118 residue is glycosylated in the original hemagglutinin protein.
