# Supplementary material for: Development of high-growth influenza H7N9 prepandemic candidate vaccine viruses in suspension MDCK cells
Source: J Biomed Sci. 2020 Apr 2;27:47. doi: 10.1186/s12929-020-00645-y (PMC7115086; doi:10.1186/s12929-020-00645-y)
Supplement: Supplementary file 5 — Additional file 5: Fig. S3. Identification of N-linked glycosylation at the N149 residue on hemagglutinin by liquid chromatography-tandem mass spectrometry. N-linked glycosylation was identified by liquid chromatography-tandem mass spectrometry, as described in Additional file 6. Tandem mass spectra (MS2) of WLLSNTDNATFPQMTK (m/z 934.44, + 2) derived from the trypsin-digested purified H7N9 bulks, (A) NHRI-RG4 and (B) NHRI-RG5. N# represents the deamidated asparagine which indicates that the N149 residue is glycosylated in the original hemagglutinin protein. [file 12929_2020_645_MOESM5_ESM.pdf]

Additional file 5

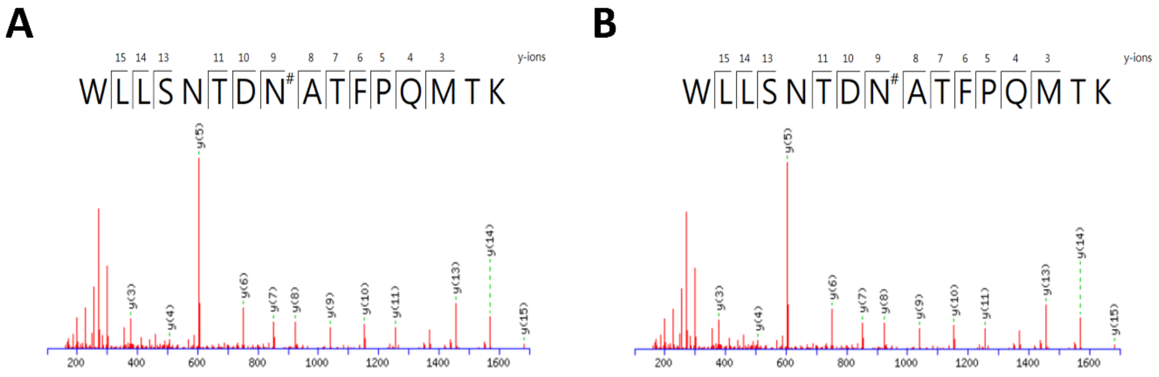

Figure S3. Identification of N-linked glycosylation at the N149 residue on hemagglutinin by liquid chromatography-tandem mass spectrometry.

N-linked glycosylation was identified by liquid chromatography-tandem mass spectrometry, as described in Additional file 6. Tandem mass spectra (MS2) of WLLSNTDNATFPQMTK (m/z 934.44, +2) derived from the trypsin-digested purified H7N9 bulks, (A) NHRI-RG4 and (B) NHRI-RG5. N# represents the deamidated asparagine which indicates that the N149 residue is glycosylated in the original hemagglutinin protein.
