# Supplementary material for: Development of high-growth influenza H7N9 prepandemic candidate vaccine viruses in suspension MDCK cells
Source: J Biomed Sci. 2020 Apr 2;27:47. doi: 10.1186/s12929-020-00645-y (PMC7115086; doi:10.1186/s12929-020-00645-y)
Supplement: Supplementary file 6 — Additional file 6: Fig. S4. Evolution of the hemagglutinin N118 glycosylation site in H7N9 viruses from the 1st to 5th epidemic wave. Temporal pattern of S118N (A) and I120T (B) mutations in H7N9 hemagglutinin from human, avian and environmental samples. HA protein sequences were collected and analyzed as described in Additional file 6. [file 12929_2020_645_MOESM6_ESM.pdf]

Additional file 6

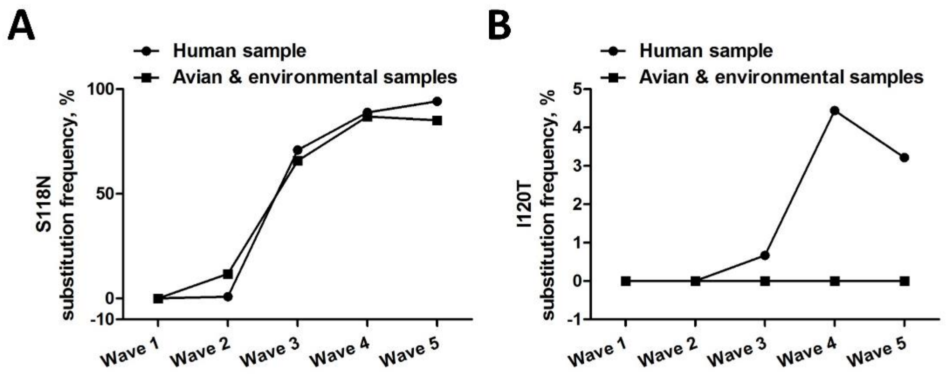

Figure S4. Evolution of the hemagglutinin N118 glycosylation site in H7N9 viruses from the 1st to 5th epidemic wave.

Temporal pattern of S118N (A) and I120T (B) mutations in H7N9 hemagglutinin from human, avian and environmental samples. HA protein sequences were collected and analyzed as described in Additional file 6.
