# Supplementary material for: Development of high-growth influenza H7N9 prepandemic candidate vaccine viruses in suspension MDCK cells
Source: J Biomed Sci. 2020 Apr 2;27:47. doi: 10.1186/s12929-020-00645-y (PMC7115086; doi:10.1186/s12929-020-00645-y)
Supplement: Supplementary file 7 — Additional file 7: Supplemental materials and methods [file 12929_2020_645_MOESM7_ESM.docx]

**Additional file 7**

**Supplemental materials and methods**

**Negative-stain electron microscopy (EM)**

The negatively stained sample was prepared as follows: 4 μL of purified viral bulk was adsorbed onto glow-discharged carbon-coated grids (Electron Microscopy Sciences), washed once with a drop of ddH_2_O, negatively stained with a drop of 2% uranyl acetate (UA) and air dried. The EM images of stained viral particles were captured using a JEM-1400 at a magnification of 60,000 and with an accelerating voltage of 120 kV. The digital images were recorded using a Gatan, Inc. Ultrascan 4000 4k x 4k Camera System (Model 895).

**Identification of amino acid substitution in H7N9 HA protein**

All HA protein sequences of laboratory-confirmed H7N9 viruses (between January 1, 2013 and October 5, 2017) were downloaded from the National Center for Biotechnology Information and Global Initiative on Sharing All Influenza Data (GISAID) databases. Duplicate or incomplete sequences were excluded from this data set, and the remaining HA sequences were aligned using Molecular Evolutionary Genetics Analysis (MEGA) 7 software.

**Identification of N-linked glycosylation by liquid chromatography-tandem mass spectrometry (LC-MS/MS)**

The method for the determination of glycosylation sites developed by Wang et al. (1) was adopted in this study. Briefly, the BCA assay was used to quantify the protein concentration of the sample, and 20 μg of the sample (total protein) was deglycosylated by treatment with PNGase F glycosidase in 50 mM ammonium bicarbonate buffer (pH 7.8) overnight at 37°C. Subsequently, the deglycosylated sample was reduced with DTT (10 mM) at 56°C for 60 min, and then alkylated with iodoacetamide (IAM; 250mM) in the dark at room temperature for 30 minutes. The denatured sample was then digested with trypsin (1:20, mass ratio) at 37°C overnight. Digested sample was analyzed by a Q-Exactive mass spectrometer (Thermo Scientific, Bremen, Germany) coupled with an Ultimate 3000 RSLC system (Dionex, Sunnyvale, CA). The LC separation was performed using a C18 column (Acclaim PepMap RSLC, 75 μm ×150 mm, 2 μm, Dionex) with a linear gradient from 1% to 35% of mobile phase B (mobile phase A: 5% ACN/0.1% FA; mobile phase B: 95% ACN/0.1% FA) for 35 min, 35% to 80% mobile phase B for 32 min and 80% mobile phase B for 10 min in a total separation time of 90 min. A full MS scan was performed with an m/z 300−2000 range, and the 10 most intense ions were selected for MS/MS acquisition, which was set to start at m/z 70. The raw data from the Q-Exactive were processed into a peak list using Proteome Discoverer 1.3. A search was performed against protein sequences of hemagglutinin (Supplementary Table 1) with the parameters including cleavage at the C-terminal lysine and arginine except for KP and RP when trypsin was used. Deamidation of asparagine (N) and glutamine (Q) was selected as a variable modification to determine the glycosylation site. Up to two missed cleavages were allowed. The intensity ratio cutoff was 10%, and mass tolerance was ±10 ppm.

1. Wang, B., Tsybovsky, Y., Palczewski, K. & Chance, M. R. Reliable determination of site-specific in vivo protein N-glycosylation based on collision-induced MS/MS and chromatographic retention time. J Am Soc Mass Spectrom. 2014;25:729-41.
